# A Manual of Medical Jurisprudence

**Published:** 1844-01

**Authors:** 


### ART. XVII.

1. *A Manual of Medical Jurisprudence.* By ALFRED S. TAYLOR, Lecturer on Medical Jurisprudence and Chemistry at Guy's Hospital. —*London,* 1843. Sm. 8vo, pp. 680.
2. *Principles of Forensic Medicine.* By W. A. GUY, M.B. Cantab., Professor of Forensic Medicine at King's College, London.—*London,* 1843. Sm. 8vo. Parts I & II.

WE regret that the absurd plan of publishing *in Parts,* adopted, in the case of the work second on our list, prevents our including Dr. Guy's incomplete volume in our present notice. Our available space will also permit us to give only a very brief account of Mr. Taylor's work. Perhaps, when noticing Dr. Guy's volume, on its completion, (of which our knowledge of the talents and learning of the author leads us to augur excellent things,) we may be able to do more justice to the very admirable treatise, which we can only introduce to our readers on the present occasion. Mr. Taylor has done injustice to himself in naming his book *a Manual;* small as the volume appears, and singularly cheap as it is, it is in reality not only the best and completest treatise on medical jurisprudence to be found in any language, but it is, in extent, one of the largest. By means of Mr. Palmer's Lilliputian type, Mr. Churchill has contrived to cheat his customers into the purchase of a huge systematic treatise, under the guise of a pretty little hand-book, and at the seductive cost of only a dozen shillings. Now we by no means object to the smallness of the price, and should not quarrel with Mr. Churchill if he sold us the book for half the sum : but verily our post-climacteric vision, aching from a long session over the alluring pages, bids us ask, whether it is really necessary thus to crowd the matter of two or three large octavo volumes into one small one? The print is clean and clear, we allow, and the paper fair and good,—and possibly the sharp-sighted frequenters of the schools and hospitals may fail to discover the typographical defects which are so painfully conspicuous in our old eyes. Possibly, also, when the publisher reckons up his thousands of purchasers among the young, he will care little for the discontent of the few hundreds of spectacled grumblers, like ourselves. But we shall have one revenge, nevertheless; and, accordingly, here warn all and sundry of our sexagenarian colleagues, neither to buy nor borrow "Taylor's Manual," but to get some of their juvenile acquaintances to read to them, from their own copies, the indispensable information it contains.

The great importance of medical jurisprudence, as a branch of medical study, is well shown in a few brief paragraphs in Mr. Taylor's preface; to which we entreat the attention of all our junior readers, whether students or practitioners:

"MEDICAL JURISPRUDENCE, whereby we are to understand *that science which teaches the application of every branch of medical knowledge to the purposes of the law,* is now so well known as to render it unnecessary for me to enter into any explanation of its objects. Its claims, as a distinct science, to the attention of the profession rest upon two grounds; 1st, that the subjects of which it treats are of practical importance to society; and 2d, that they are not included in the other branches of a medical education.......

"It is unnecessary for me to remark that great responsibility is attached to the duties of a medical witness, and that any member of the profession may find himself involved in this responsibility, from circumstances of a merely accidental nature. When a crime, requiring medical evidence for its elucidation, is perpetrated, the duty of the whole investigation commonly devolves on the practitioner, who lives nearest to the spot; it is therefore virtually upon his knowledge and experience, that the clear proof of the crime and the legal punishment of the offender, must rest.......

"Some medical practitioners are disposed to treat medico-legal inquiries with indifference. They are apt to think, that cases are rare; and that they may easily escape any grave responsibility when they occur. *I have never known this indifference manifested by one, who had once been summoned as a medical witness to a court of law;* and as to the rare occurrence of cases, I may perhaps be permitted to make the following remarks. From some recent Parliamentary returns, it appears that in one year in the United Kingdom, there were 1213 trials involving questions of murder and manslaughter, either perpetrated or attempted from POISONING and WOUNDS alone, in every one of which medical evidence was necessary, and in the majority, indispensable to conviction. In two years, there were 541 deaths from poison in England and Wales alone, in the greater number of which, medical evidence was absolutely required. This is exclusive of criminal attempts at poisoning not followed by death." (Pref., pp. 5-9.)

The shrewd remark in the last extract, which we have put in italics, contains a most forcible *argumentum ad hominem*, which every young practitioner would do well to listen to; and we can honestly say we know of no means so likely to fortify him against the distressing contingency hinted at, as the attentive study of Mr. Taylor's book.

The plan and arrangement of the contents of this volume are entirely practical, and sufficiently indicate that the author has fully profited by his long experience as a teacher and medical jurist. In this respect, as in many others, the volume differs from all its predecessors.

"The only arrangement which it has appeared to me advisable to follow, has been that of placing the subjects in the order of their importance. Thus POISONING, WOUNDS, and INFANTICIDE, constitute more than three fourths of those cases which require the aid of a medical jurist in a court of law. Hence these have been treated at a length commensurate with their importance. The subjects are examined in the form of questions, and those on TOXICOLOGY, more especially in reference to general poisoning, have been framed, with some modifications, on the plan originally proposed by Orfila and Christison. The arrangement of the questions, relative to WOUNDS and INFANTICIDE, has been chiefly derived from an analysis of the medical evidence given on the numerous trials connected with those crimes during the last fourteen years. By this arrangement, it is expected that every point of any practical interest will be brought before the reader. A large number of the cases in this Manual have never before been published. They are entirely derived from modern sources, and it is anticipated that these will be much more serviceable to the practitioner as illustrations of the duty required of him, than those drawn from old and obsolete annals. Some of these cases I have been able to collect by my connexion with Guy's Hospital,—many have been kindly furnished to me by those gentlemen, who, in the course of the last thirteen years, have attended my lectures on Medical Jurisprudence in that institution; and others again I have derived from the quarterly and weekly medical periodicals of England, Scotland, France, and Germany. Where the limits of the subject would not allow me to introduce illustrative cases, ample references have been given, so that the medical or legal student, who requires further information on particular questions, may easily obtain it." (Pref., pp. 5, 6.)

The following is a brief synopsis of the whole contents:

| | | | | |
|---|---|---|---|---|
| I. | Poisoning . . . | treated in | 27 | Chapters. |
| II. | Wounds . . | ,, | 14 | ,, |
| III. | Infanticide . | ,, | 8 | ,, |
| IV. | Death from drowning . | ,, | 1 | ,, |
| V. | Hanging . . | ,, | 1 | ,, |

| | | | | |
|---|---|---|---|---|
| VI. | Strangulation . | treated in | 1 | Chapter. |
| VII. | Suffocation . . | ,, | 2 | ,, |
| VIII. | Lightning, cold, starvation | ,, | 1 | ,, |
| IX. | Rape . . | ,, | 1 | ,, |
| X. | Pregnancy, delivery | ,, | 2 | ,, |
| XI. | Birth, inheritance . | ,, | 1 | ,, |
| XII. | Legitimacy . | ,, | 2 | ,, |
| XIII. | Insanity . . | ,, | 4 | ,, |

The practical character of Mr. Taylor's treatise has led him to give only a subordinate place to several topics which other authors have dwelt largely on. Although we entirely agree with the author as to their small relative importance, we still think it would have been useful, for the sake of more convenient reference, to have given to each of these subjects a separate consideration. These are Mr. Taylor's reasons:

"With respect to FEIGNED DISEASES, the duties of a practitioner rarely go beyond the detection of feigned poisoning, wounds, pregnancy, delivery, and insanity; and an account of these will be found in the chapters on those subjects. The practical points connected with AGE, IDENTITY, and SURVIVORSHIP, as they concern a medical witness, will be found scattered throughout the volume. In almost every case of AGE or IDENTITY the question is settled by the evidence of non-professional witnesses; and it has therefore seemed to me improper to introduce distinct chapters upon such subjects to the necessary exclusion or abbreviatiou of other cases which are of much greater practical importance and more frequent occurrence." (Preface, p. 7.)

To give anything like an analysis of this work would require ten times the space that we can now afford; and, to say truth, we would hardly attempt such a task even if we were unhampered in this respect. This is not one of those sappy and windy productions which the industrious analyst concentrates into an essence for the benefit of the indolent or fastidious; nor one of those whence the cunning critic may extract a spicy kernel from its mountain of husk: it must be had in its own bodily form, must be read, studied, and kept for reference. Wherefore we consider that we shall best fulfil our duty to our readers by limiting our present labours to a concise exposition of what they will find in Mr. Taylor's book.

Chapter I is, we believe, new in works on medical jurisprudence. It involves the consideration of what substances should be regarded as poisonous and what not, a point on which medical witnesses have differed much on trials for poisoning. The subject is of some interest in relation to death from over-doses of saline medicines, commonly reputed inert, as in respect to salt (p. 2), sulphate of magnesia (p. 3), and sulphate of potash (pp. 131 and 594.) The late case of the Queen v. Haynes is, in this respect, of some importance. The medico-legal facts connected with mechanical irritants are also fully stated. (p. 8.)

In Chapter II, "On the mode of action of poisons," the author has followed Orfila and Christison. An experiment is related at page 15, where ferrocyanate of potash was detected not merely in venous blood, but in the contents of the thoracic duct. We think that the facts connected with the detection of poisons in the blood, are more recent and more fully and practically stated than in either Orfila or Christison.

In Chapter III, the author has followed Dr. Christison in adopting the classification proposed by Orfila, but has formed more practical subdivisions of the irritant poisons. He has also here set down, in a few simple tables, the names of all those substances which have either destroyed human life, or have given rise to serious symptoms. (p. 24.)

Chapter IV, "On the rules to be observed in investigating a case of poisoning," seems to us entirely new in its plan, in works of this kind. It cannot fail to be very useful in obviating the difficulties experienced by young practitioners on first entering on the medico-legal investigation of poisoning.

In Chapters V, VI, VII, VIII, the mode of examining the evidence of poisoning is detailed. The plan of these four chapters has been derived from Orfila, who in this was followed by Christison; and it does not appear that any better plan could be devised. The author's opinions are deduced from the occurrence of actual cases, many of which are new, and are now reported for the first time in a work on medical jurisprudence. They are accompanied by comments to show their practical bearing.

Chapters IX and X present some novelty in the mode in which the subject of poisoning is therein treated. In Chapter X are some valuable statistical tables, condensed and arranged, showing the frequency of the occurrence of cases of poisoning, (p. 83;) and what are the substances which specially claim the attention of a practitioner. Tables of this kind have not, to our knowledge, before appeared in any medico-legal work.

With respect to the chapters on the Poisons individually, the author has classified the main medico-legal questions in distinct paragraphs. Most medico-legal writers have done this with respect to symptoms and treatment; but here will be found described in addition, under every poison, 1st, the *quantity* required to destroy life, the *smallest* quantity which has killed, the *largest* quantity from the effects of which a person has escaped; 2d, the period within which each poison commonly destroys life, including the *shortest* fatal period, and the *longest* fatal period known. Dr. Christison has followed the same plan with respect to two or three poisons; but his remarks are on these points very limited, (we say this without imputing any blame to him,) and are mixed up with other subjects. Mr. Taylor has here collected a vast number of cases, which had not even occurred when Dr. Christison published his earlier editions.

The chemical analysis of each poison is very full, and the objections to each test, and the answers to the objections are carefully pointed out, —a circumstance hitherto but little attended to in medico-legal treatises. Chemical evidence has often failed, simply because a medical witness could not satisfactorily answer the objections which were made to the tests.

In the chapters on "General poisons," the author has shown that they may be detected in organic matter after many years' exposure to decomposition,—in the case of sulphuric acid, for twelve years; of arsenic, for nine years, &c. &c. To each poison is appended an account of the method of determining the *quantity present* in a given mixture, a most important medico-legal question, but, strange to say, wholly omitted by those otherwise excellent authorities, Orfila and Christison.

Chapter XX (p. 216) is, we believe, new in medico-legal toxicology; it presents the arrangement of the principal poisons in tables, so as to show, at one view, the results of numerous selected tests, whereby the nature of most poisons may be determined in a few minutes. Orfile published some analytical tables a few years ago; but they were complicated and defective, and more chemical than toxicological. The present tables cannot fail to be of the greatest service to practitioners.

The second great subject, "Wounds," is treated in a masterly manner in the next fourteen chapters. The arrangement is evidently derived from practical observation of what is most important in cases likely to become the subject of legal investigation. Like all the other subjects, it is illustrated by a great number of original cases. We would call the attention of our readers, in a particular manner, to the excellent account given of the value of *blood-stains* as evidence, in Chapter XXXIV; and to the observations in regard to *locomotion*, &c., after wounds, in Chapter XXXIII. These contain numerous very interesting facts and conclusions of the highest importance, which have not hitherto met with the consideration they deserve. The same remarks apply to Chapter XLI, "On gunshot wounds," which is full of the most interesting details, never before published in any work of this kind.

The third main subject of the work, "Infanticide," is comprehended in eight chapters; which, in our judgment, leave nothing of any importance unnoticed, and lay down the soundest views of disputed points.

In Chapter LII, on "Hanging," among other interesting matter, will be found some important facts connected with the proofs of hanging in the living and dead subject, founded on Casper's experiments, published some time since in this Journal.

Chapter LIV, on "Suffocation by carbonic acid," is also of especial interest and importance; and contains many original experiments and observations. (p. 555.)

Chapter LVII, on "Rape," and Chapter LX, on "Birth and inheritance," involve some new and curious medico-legal points.

The four chapters on "Insanity" comprehend all the medico-legal facts relating to that most important subject; one chapter (LXV) being devoted to the consideration of responsibility for crime, and the medical and legal tests of insanity.

In an Appendix we have (A) a list of the tests and apparatus required in poisons; and (B) an abstract of the provisions of the Medical Witnesses' Act before Coroners; both of which are likely to be of great service to a practitioner.

In conclusion, we can conscientiously say of this book, that it presents, in our judgment, as complete and perfect a view of the whole subjects of which it treats, as the present state of our knowledge and the extent of the allotted space admit of. Everywhere the author demonstrates his practical familiarity with his subject; while the conclusions come to on all disputed points, seem the natural products of a masculine understanding and sound judgment, tested and chastened by a long experience. As well in the matter of materials, as in the manner of treating many of the questions, there is much more originality in this work than might be expected, either from the general subject of it or from its unpretending form. Through it the author has assuredly entitled himself to be considered as one of the most accomplished medical jurists of present or former times. It would be superfluous, after such praises, to recommend, in more formal terms, the 'Manual of Medical Jurisprudence' to the attention and careful study of all our readers.

